# What are the impacts of setting up new medical schools? A narrative review

**DOI:** 10.1186/s12909-022-03835-4

**Published:** 2022-11-07

**Authors:** Ferhana Hashem, Catherine Marchand, Stephen Peckham, Anna Peckham

**Affiliations:** Centre for Health Services Studies, University of Kent, George Allen Wing, Cornwallis Building, Kent, Canterbury CT2 7NF UK

**Keywords:** New medical schools, Impact, Health outcomes, Social accountability, Workforce, Local economy, Research activity

## Abstract

**Background:**

The growth of the UK’s population together with an aging society with increasingly complex health and social care needs has placed a greater demand on statutory care services. In view of this emerging landscape, the UK Government has sought to increase its medically trained workforce in order to better respond to the demands placed on the health service. Five universities were announced as homes to new medical schools offering undergraduate places to boost the numbers of doctors training in England. The aim of this narrative review was to explore how new medical schools could improve the health outcomes of the local population and evaluate the potential contribution it may make to the local economy, workforce and to research and innovation.

**Methods:**

A narrative review was undertaken using a systematic approach for the search literature strategy. The articles were evaluated by undertaking a critical assessment evaluating the fitness of a paper for review according to results, methods used to test the hypothesis, conclusions and impact and limitations. Thematic analysis was employed to organise and summarise the findings across a heterogeneous body of literature included in the review. The analysis was developed in an inductive manner and there were not any predefined themes to guide data extraction and analysis.

**Results:**

Thirty-six articles were selected for inclusion for this narrative review. The review identified six key themes: influence of prior rural exposure, medical school environment and rural enrichment programmes, workforce, health outcomes of local populations, social accountability, economic contribution of medical schools to communities and impact on rural research.

**Conclusions:**

The studies included found a wealth of information on a wide-range of topics on the expansion of undergraduate education and its implications on the future medical workforce. It was shown that medical schools can have a positive effect on the health, social, economic and research activity of a region, but this literature tended to be heterogeneous in focus without consideration of the inter-connections between the wider societal and economic impacts arising from long-term sustainable change being brought to a region.

**Supplementary Information:**

The online version contains supplementary material available at 10.1186/s12909-022-03835-4.

## Background

The growth of the UK’s population together with an aging society with increasingly complex health and social care needs has placed a greater demand on statutory care services [1, 2]. The UK’s National Health Service (NHS) has grown and expanded to support these demographic and epidemiological trends offering a seven-day service evolving a model of delivering care that is more person-centric and integrated across organisational and sectoral boundaries [3]. In view of this emerging landscape, the UK Government has sought to increase its medically trained workforce in order to better respond to the demands placed on the health service. Despite removing the limits on the numbers entering some healthcare professions namely nurses, midwives and allied healthcare professionals in 2017, the restrictions on the numbers of students entering medical schools have remained unaltered [4]. The issue is further compounded by the system of medical education being remarkably competitive, even though medical schools in the UK receive a significant number of applicants, each year medical schools refuse applications from individuals who have the potential to contribute to a career in medicine. In 2017 (prior to the announcement of the extension of medical school places to students in 2018) there were 20,000 applications to medical schools, with only 8000 places being available in the whole of the UK [3, 5]. Areas of medicine struggling to recruit graduates are in general practice and in many other specialities (such as psychiatry) in a number of regions not exclusively but including rural, coastal and urban areas [4].

The expansion of medical school places was put forward by the UK Government in 2016 as a potential solution to expanding the medical workforce with a proposed increase of 1500 places, which would be comprised of 500 additional places for allocation across existing medical schools and a further 1000 places in new medical schools [5]. The context underpinning the expansion was driven by a recognition in UK Government policy that in order to meet the growing challenges of people living longer requiring more support from health and social services, alongside evolving models and integrated care for people with more than one-term condition, as well as a greater focus on community services to avoid hospital admissions, the current medical workforce needed to be expanded to better respond to the demands placed on the UK’s National Health Service (NHS). To meet these challenges, according to UK Government policy, increasing the numbers of doctors trained was regarded as the most cost- efficient way to allow medical schools to expand in a managed way [3]. A competitive selection process was undertaken and in March 2018 five universities were announced as homes to new medical schools offering undergraduate places to boost the numbers of doctors training in England [6]. Areas for new medical schools were selected in Sunderland, Chelmsford, Canterbury, Lincoln and Lancashire, with Anglia Ruskin Medical School kick-starting enrolment of undergraduates for a 2018/19 intake [7]. By 2021, there were 9000 medical school places following the Government’s adjustment to the cap on numbers. The new medical schools were chosen in areas with systemic staff shortages in medicine and difficult to fill vacancies [8]. It has been shown in research by Goldacre et al. 2013 in their cohort survey of 31,353 UK trained doctors in 11 cohorts from 1974 to 2008, doctors were more likely to work in the region they trained in, with 48% undertaking specialty training in the same region as their medical school. In addition, 34% of respondents who had reached GP or consultant status has settled in the same region as their home [9]. There were similar patterns of medical graduates meeting the workforce needs of a region seen in the first six cohorts graduating from James Cook University (JCU) from 2005 to 2010 in Queensland, Australia. Most graduates had accepted undertaking their internship in Queensland and we less likely to want to work in metropolitan areas, preferring to stay in outer regional and rural centres [10]. Doctors therefore invariably were more likely to work in areas in which they trained, therefore the aim of the five new medical schools is to retain and recruit doctors in areas which have traditionally been underserved by a medical workforce [7, 11].

This paper is embedded within an English medical education policy context and it is vital to acknowledge the historical vestiges associated with the establishment of new medical schools in the UK. In the decades prior to this most recent period of expansion, from 1997 to the mid-2010s, the establishment of new English medical school was shaped by three key concerns: a direct result of government policy to increase and control the number of medical students, an opportunity for the universities to undergo some expansion and for the GMC to regulate and assert its authority with respect to pedagogy [12]. Yet, as the UK moves towards the mid-part of the twenty-first century, it is important to take into account the significance of international expansion models, as seen in Australia, Canada and the United States, with correspondingly similar issues encountered around recruitment and retention of the clinical workforce, barriers to accessing appropriate healthcare and insufficient understanding about health care needs in a non-urban context. With a global trend of increased urbanisation with a move away from rural to urban dwelling, concerns around workforce development in underserved regions require further knowledge exchange and learning across national boundaries for future policy planning [13].

The impact of a new medical school to a region goes far beyond an increase in medical school places with the ensuing increase of the medical workforce [14], but has the potential for bringing widespread change to aspects of a region’s resources, commercial interests, economy, health and research activity. The social and economic impacts considered in this paper concentrate around four related benefits which have the potential to transform a region: (1) economic sustainability; (2) improvements in the social determinants of health and health equity; (3) addressing social accountability of the medical school in the region; and lastly, (4) increasing research activity [15, 16]. With respect to economic sustainability, new medical schools encourage people to live, work and learn in communities that are economically challenged [11]. In Hogenbirk et al. 2021’s recent research on the Northern Ontario School of Medicine (NOSM) in Canada, it was found that for every dollar spent by NOSM in support of the medical education programme and associated activities including spending by staff, clinical teachers and learners, an estimated $0.66 cents (CAD) was generated in additional economic activity in 2019 in NOSM’s service region of Northern Ontario. The economic impact in Northern Ontario increased by 60% over 11 years from $67 M CAD in 2008 to $107 M CAD in 2019. However, often the wider community impacts are still unknown. Hogenbirk et al. 2021 suggest in addition to an increase in expenditure on associated economic activity, there is the potential to improve the social determinants of health and the health of the population [11].

Moreover, when the new medical schools were announced in England, a driving force behind the expansion was not only to address national recruitment, but provide clinical placements in specialties to regions where the shortage of doctors is the most acute. A key aspiration is that the new medical schools would be placed in areas based on the availability of clinical placements and ultimately around the needs of the local populations within geographical areas [4, 5]. Regionality is a critical factor – not only providing clinical placements in large urban teaching hospitals, but instead distribution of placements would focus on building capacity in rural and coastal settings and smaller hospitals serving local populations. In the UK the ‘rural’ context is largely linked to this concept of regionality as a key justification for recruiting from and serving underserved regions [4]. This shift in placements and learning opportunities, it is hoped, will help to re-focus expansion efforts of medical schools on under-doctored regions and specialties [4]. Sen Gupta et al. 2013 have shown how the growth of rurally orientated medical schools and placements in Queensland, Australia has led to a growth of postgraduate pathways in formal general practice training, with trainees completing their training through a geographically dispersed team of educators, who provide careers advice, are advocates for the trainees and assist with negotiating new posts [17, 18]. The trend seen in Queensland suggest there is the prospect of medical graduates training regionally addressing the workforce needs of under-doctored regions and specialities in the new English medical schools.

Medical schools have a responsibility to operate under a social accountability framework in their region. The notion of the social accountability of medical schools was first introduced by the World Health Organisation in 1995, which defined medical schools as having, “the obligation to direct education, research and service towards addressing the priority health concerns of the community, region, and/or nation they have a mandate to serve” [19, 20]. The concept has been further refined in a statement in 2010 by the *Global Consensus for Social Accountability of Medical Schools* [21] and the 2017 World Summit on Social Accountability [19], which has now recognised the contribution medical schools make to engaging, partnering with and responding to the needs of underserved and vulnerable populations [19]. There are significant opportunities to form community collaborations between medical schools and regional health care organisations to improve the education, research and healthcare for an entire region to the benefit of the population [13].

Lastly, of particular interest to universities introducing new medical schools is the research potential to the region, along with local enterprise companies, pharmaceuticals and local government being acutely aware of the substantial and increased research funding opportunities available. According to Catto (2000), biomedical and pharmaceutical research is likely to be sourced not only exclusively by public funds per year, but includes an estimated 10% of pharmaceutical research expenditure being available to universities [16]. Joint infrastructure funding is critical to help strengthen research facilities and equipment required for innovative studies in biomedical sciences in which the UK Government and the Wellcome Trust play a leading role. High quality research undertaken by well-trained researchers should have a positive effect on the retention and motivation of staff [16]. Furthermore, there is a growing body of evidence to suggest that improved research activity not only has academic benefits but is directly associated with better patient outcomes [22, 23]. Embedding research into healthcare both drives high-quality patient care and is highly rated by participating staff and patients further reinforcing the relationship between research and quality of care [24].

Our narrative review focused on what contributions new medical schools bring to a region with respect to their wider health, social, economic and research impacts. New medical schools are not only tasked with developing new doctors, but training doctors to explicitly tackle health inequalities in underserved communities and delivering relevant patient care to those communities. We chose a narrative review to synthesise the evidence deeming it an entirely suitable method to review a combination of different study types and providing a reflective lens to help deepen an understanding of the contribution new medical schools make. A narrative review does not aim to solve a problem or puzzle requiring data, but is undertaken to help formulate a view, insight or point for clarification in which a more interpretative and discursive synthesis of the literature is needed [25]. Furthermore, a narrative review can address one or more question and the selection criteria for inclusion or exclusion may not be explicit. Despite no consensus on the standard structure of a narrative review and no acknowledged guidelines available [26], this type of review can benefit from the same methodological rigour of a systematic review, include defining the key issues, providing clear inclusion and exclusion criteria for a literature search, narrowing the focus on a set of studies and including a relevant criteria of reviewed studies [27]. Examining the indirect outcomes of new medical schools is a key motivation for our review with respect to the health impacts, economic contribution, social effects and new opportunities arising for research.

## Methods

A narrative review follows no formal standard structure or available guidelines, but they should respect the preferred format which is IMRAD (Introduction, Methods, Results, and Discussion) presenting the results in either a chronological format or as a conceptual or thematic framework separated around dependent or independent variables [26, 27]. The structure developed by Ferrari (2015) building upon Green et al.’s (2006) work on writing narrative reviews was used and we used a systematic approach for the literature strategy to identify key concepts and gaps in the literature [26–28]. The articles were evaluated by undertaking a critical assessment, as suggested by Ferrari (2015), evaluating the fitness of a paper for review according to results, methods used to test the hypothesis, conclusions and impact and limitations [27].

### Aims

The aims of the narrative review were to explore the contribution of new medical schools to the health, social, economic, and research impacts to a region, and consider what the implications are on the workforce. The wide-ranging aims and scope of the review required consideration of a fairly broad and diverse range of literature, and two research questions were developed specifically to answer the question.

### Research questions

1. What is the contribution of new medical schools on the health, social, economic and research impacts in a region?
2. How do new medical schools effect workforce issues in the local economy?

### Literature search

A literature search was undertaken to identify relevant published papers from three electronic databases, this included: EMBASE, Medline (Ovid) and Web of Science Citation Index. The search strategy was developed after an initial pilot search of Medline and adapted for the other two databases (please refer to Table S1: Medline Search Strategy), and was undertaken with the assistance of a research librarian. The search was performed in February 2021. The literature search involved a combination of text terms, keywords and MeSH subject heading terms given in Table 1. The authors devised a list of inclusion and exclusion criteria which are given in Table 2.

**Table 1:** MeSH terms and Keywords

PICO(s)	Keywords
Population	Rural or proximal population
	Local citizen
	Patient
	Medical students
	Healthcare Workforce
	Health professionals
	Trusts
	Pharma companies/ organisations
	Research, researcher* (Local)
	Hub*
	Primary Care Network
Study design	Evaluation
	Quantitative
	Qualitative
	Review*
Outcomes	Service* in the community (A&E, maternity ward, etc.)
	Option for future potential patents
	Recruitment of healthcare professionals
	Retention of healthcare professionals
	Patient care (improvement)
	Local population health (improvement)
	Economic impact
	Biomedical impact
	Health economic impact; regional health improvement
	Sustainability

**Table 2:** Inclusion and exclusion criteria

Inclusion criteria	Exclusion criteria
Papers published in English and French	
New medical schools and rural hubs	Not medical education
Community-based medical schools	Not postgraduate education
Primary-care based medical schools	
Undergraduate medical curriculum	
Widening participation initiatives	
Early clinical contact	
Placement*	
Included date from 2011 to 2021	

The authors devised a list of inclusion criteria for the search which included a date range of between 2011 to 2021, review articles, countries with similar healthcare systems to the UK and English and French language papers, as well as articles of individual studies examining the wider contributions on the health, social, economic and research impacts of medical schools. The exclusion criteria were: not medical education and not postgraduate education.

### Critical assessment

Data was extracted from each article on the (1) type of study, (2) methods used to test hypothesis, (3) results (4) conclusions and impact, and lastly, (5) limitations. The data was collected using tables in MS Word which made it efficient and easy to extract the relevant information and undertake a preliminary synthesis. For each paper, the author(s) names and dates were included in a column down one side of the table, with the categories for data extraction along the top. The information in the table was then used to compare all of the data extracted against the full text of the original papers.(26, 29).

The preliminary synthesis was translated into thematic analysis in order to identify the main recurrent themes and concepts across the variety of papers. Thematic analysis was employed to organise and summarise the findings across a heterogeneous body of literature. Using the data extracted from the tables, the thematic analysis involved focusing on similar topics that had conceptual overlap; even if apparently similar concepts were not overly explicit from the results, the main concepts were ‘interpreted’ and used to explore similarities and differences between different studies [29]. The analysis was developed in an inductive manner and there were not any predefined themes to guide data extraction and analysis. A rigid a priori conceptual framework had not been preconceived or framed around predefined concepts. Nevertheless, without an inductive and deductive interaction centred upon data analysis, the analysis produced would have been inappropriate and irrelevant, therefore it is acknowledged that the authors had prior knowledge of the relevant literature which inevitably impacted on developing the theoretical boundaries informing the themes [30]. The thematic analysis reflected and connected different findings under a theme, but the main ideas and conclusions did not generate any new knowledge from the papers reviewed [29].

## Results

### Study selection

**Fig. 1:**
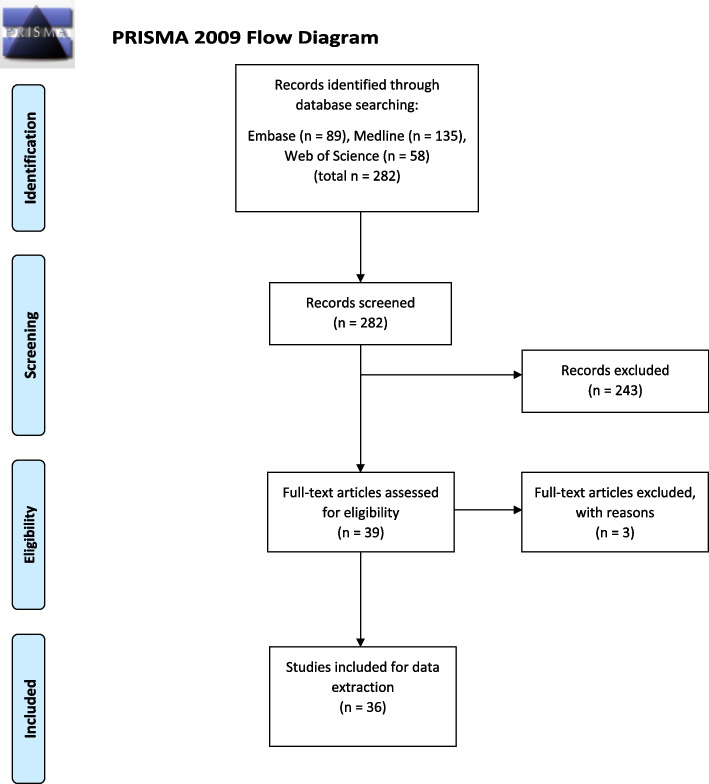
PRISMA Flow Chart

The search was limited to materials published in English and French from 2011 to 2021. The search included review papers and government reports. Despite the search including French language papers, no papers were found. After an initial search, the three electronic databases yielded 282 results studies after duplicates were removed. The title and abstract of 282 papers were screened and 243 were excluded. Overall, full-text articles of 39 papers were read in full with a further three excluded. Three papers were excluded due to being out of scope. Papers were checked for eligibility against the original inclusion and exclusion criteria which was applied against all papers subjected to a full-review. Ultimately, 36 articles were selected for inclusion for this narrative review (see Fig. 1: PRISMA Flow Chart).

### Study characteristics

Of the articles included in the review, the majority originated from the USA (*n* = 10; 27.8%), followed by Australia (*n* = 8; 22.2%), Canada (*n* = 7; 19.4%), UK (*n* = 3; 8.3%) and Germany (*n* = 2; 5.6%). Single published articles originating from one country were from: Chile, Indonesia, Japan, Nepal, South Africa and Switzerland. Twenty-two articles were evidence reviews including systematic reviews, narrative reviews, an integrative review, critical reviews and scoping reviews. There was one survey paper, one quantitative study, one qualitative study and a combined qualitative literature review/ mixed methods article. Included were eight review articles on educational policy, government strategy and workforce data. There were two opinion / perspectives articles. Table S2 provides a summary of the sources that were included in the review.

### Key findings

The literature review has identified six key themes: influence of prior rural exposure, medical school environment and rural enrichment programmes; workforce; health outcomes of local populations; social accountability; economic contribution of medical schools to communities, and lastly the impact on rural research.

#### Influence of prior rural exposure, medical school environment and rural enrichment programmes

The literature on undergraduates’ and postgraduates’ career choices in primary care within a rural setting is evidenced by studies and reviews emphasising the importance of personal attributes such as rural identity [31–33] or urban origin students with a premedical school mind-set to practice rurally [34]. One study investigated whether family medicine as a specialty choice could be predicted when recruiting and admitting students to rural medical education. Avery at al [35]. found that statements of interest, plans and decisions regarding family medicine, alongside consideration of attachment to serving and living in a rural area were important factors to be elicited at the time of admission interview [35]. Students’ rural experiences in medical training have been highlighted as an important consideration informing their career choices [36]. The contribution of a ‘preceptor’ (a clinician with whom the medical student is working with and is responsible for the students’ learning) has been suggested by Stagg et al. [37] has a much stronger influence on career choices than placements [37]. Whereas in Amin at al.’s [38] rapid literature appraisal, evidence suggested that medical students can be encouraged to choose a career in primary care if they undertake a community placement of sufficient quality, quantity and duration [38]. Hurst [39] argues that despite efforts to increase medical graduates’ choices to work within primary care and in rural practice, the tendency of medical training to be highly-specialised and hospital-based turns them away from this career choice [39]. In Darbyshire et al.’s [40] systematic review of interventions on encouraging careers in medicine, it was noted that strategies tended to be focused on postgraduate training with limited amounts of evidence on undergraduate interventions [40].

Three evidence reviews demonstrated the connection between rural exposure during undergraduate medical training, and the increase in the number of medical graduates working in rural settings. Interventions noted have included attending a rural campus or spending time in a rural area to practice in non-metropolitan areas [41], short rural assignments to complete medical training in geographically remote medical schools, rural internships [42] and intensive rural health programmes [43]. One review by Guilbault and Vinson found that clinical training in rural underserved areas increased the likelihood to practice in primary care in underserved areas by about four times [44]. A fourth review by O’Sullivan et al.’s [45] on the outcome and characteristics of Australia’s rural immersion programmes in fact found that there was only a moderate association with an increased rural supply of early career doctors through rural exposure [45].

Interventions aimed at increasing primary care and general internal medicine graduates in non-rural settings in Sacramento (USA) through the Transforming Education and Community Health (TEACH), have seen a steady increase in the number of graduates who have been in practice for more than 1 year, with 35% practising in underserved communities [46].

#### Workforce

With respect to tackling problems around increasing the workforce, Danish et al. [47] noted that the establishment of medical schools was not only one of the most effective strategies to reduce shortages in doctors, but had much wider impacts regionally effectively ‘medicalising’ an area and in part improving infrastructure. Noted developments Danish et al. observed included partnerships between medical schools and local health systems, community engagement in health care, and acquisition of equipment and technological and human resources advances [47]. The return on investment in funding for health education programmes is a significant consideration for national and local stakeholders, communities and businesses when considering setting up new educational programmes in healthcare, which has been discussed by Palsdottir et al. in their study [48]. They argue that health professional education, alongside community-based / engaged learning, provides a return-on-investment in health improvements in a region. Fundamentally, it attracts and retains a health workforce and remains a solid source of job creation and contributes to economic outputs [48]. Importantly, having a fit for purpose workforce with a strong community focus is core, according to Palsdottir et al., which can help to progress community health towards achieving health equity [48]. Kirch et al. [49] also note the importance of understanding the supply and demand equations of physician workforce shortages predicted in the United States in order to drive up the health workforce. They argue that health workforce shortages have significant implications regarding physicians’ ethical commitment to ensure access to healthcare, through the expansion of medical education, without which would impact most heavily on vulnerable populations [49].

The utility of the evidence reviewed on workforce recruitment and retention issues in primary care/rural settings tended to be of limited value. It largely focused on identifying factors aimed at encouraging rural community medicine such as personal influences, medical training, postgraduate practice [50–52], and addressing primary care physician shortages or misdistribution [52–56] rather than outward facing community engagement initiatives, socio-economic repercussions or economic impacts of new medical schools.

#### Health outcomes of local populations

The establishment of rural academic centres in Australia initially set up to recruit and train rural medical students have reportedly had an impact that goes above and beyond the immediate objectives for expanding rural medical education. Lyle et al. [57] have noted in rural, regional and remote communities in Australia, rural health and rural clinical schools have created opportunities for students to engage with local communities and contribute to service delivery, and offer or partner with rural communities on local and regional health-related projects. They noted how rural health multidisciplinary training programmes have helped to improve Australian Aboriginal and Torres Strait Islander health including embedding their health issues into rural curricula [57]. Longitudinal community inter-professional student-run home visits programmes in Singapore for chronic disease and management of existing conditions, were reported by patients to have improved their health over a 6 month period indicating that students were able to assist in addressing their health issues [58].

Community-based medical education has been at the forefront of providing free health services to ‘underserved’ or deprived communities in the United States. Sandhu et al. [59] have noted how community-based medicine enables students to be immersed within the community of their practice. In some cases, students run free clinics where health professional students offer free health services under the supervision of a licensed health care professional, or in other cases ‘street clinics’ to people without medical insurance, helping to reduce barriers to accessing healthcare and potentially improving health outcomes [59]. The role of Academic Health Centres (AHCs) (in Germany, USA and Australia) was thought to be an important asset to a region to improve health equity through the delivery of health professional education. Yet, in Edelman et al.’s systematic literature review, they found that although there was substantial interest for AHCs to take on and promote health equity, they saw a profound lack of evidence to demonstrate the contribution AHCs can make towards this goal [60].

#### Social accountability

New medical schools encounter growing pressures to demonstrate *social accountability* policies in their mission statements, programmes, objectives and strategic plans [61]. Forms of accountability focus in particular on medical schools producing competent graduates prepared to respond to the changing public health care needs within their local communities, as defined by WHO and re-affirmed in 2010 in the *Global Consensus for Social Accountability of Medical Schools*, emphasising the need to produce socially accountable evidence [61]. Barber et al.’s narrative review proposes a model for evaluation that takes into consideration (a) *Context* (b) *Processes* (c) *Products* (d) *Impacts* (CIPP model) which may lead to the creation of indicators to allow institutions to monitor and progress goals and objectives in order to demonstrate social accountability, but there is little published evidence on the implementation of this framework [61]. Documented evidence of the social accountability mandate is present in the case of the Northern Ontario School of Medicine (NOSM) opened in 2005, which was developed through a community consultative process, underpinned by the region’s health context, and a holistic cohesive undergraduate curriculum connecting learning and assessment around five themes into a single integrated curriculum (https://www.nosm.ca/education/md-program/) [62]. The economic impact of NOSM is measurable, and is discussed further below [63]. Despite the importance of social accountability with medical schools embracing it as desirable and necessary, this mandate has more often than not been aspirational rather than based on tangible activity or evidence. In Ellaway et al.’s [64] critical scoping review, they examined how social missions of medical schools translate into admissions policies, and found a paucity of action or reporting outcomes from connecting social missions with admissions [64]. In Puschel et al.’s [65] study of social accountability of medical schools in academic primary care training in Latin America, they found that academic groups recognised the strong responsibility of medical schools in reducing health disparities, however, actual strategies aimed at reducing health disparities in medical schools was not evident. The lack of evidence on the social accountability of medical schools suggests that a much broader investigation is required taking into consideration system-wide and ecologically driven research to evaluate the impact.

#### Economic contribution of medical schools to communities

The evidence on the economic contribution of medical schools is somewhat limited with only one study by Hogenbirk et al. [63] looking at the case of NOSM and its economic contribution. Hogenbirk et al.’s study involved economic modelling and a focus on the broader socio-economic impacts of how new medical schools translate into both substantive economic activity, as well as providing positive socio-economic benefits to participating communities for a region as a whole. With respect to economic activity associated with NOSM, Hogenbirk et al. suggested looking at contributions on (a) *actual spend* (b) *based in estimated spending* and (c) *broader socio-economic effects*. The latter was gleaned through interviews with physicians, hospital managers, other professionals (i.e. involved in construction and renovations), senior business leaders, community leaders and local government leaders. The results found that the impact of medical education extended beyond the production of doctors and other health professionals, and contributed to NOSM’s annual income estimated at $67.1million (2007/08) with total annual income in other communities in northern Ontario of $10million. The interviewees indicated that the economic effect of the new medical school was much greater than the dollar value as it opened up new economic opportunities identified as an actual or incidental consequence of NOSM’s activities.

#### Impact on rural research

The establishment of rural academic centres have led to the growth in rural health research activity, which in turn has made a valuable contribution to targeted research serving the unique needs of rural residents, whilst at the same time better informing rural health policy [66]. In Bailey et al.’s PubMed review on Rural Clinical Schools (RCS) research in Australia, they found that there was an increase in Australian Rural Health (ARH), with Rural Clinical Training and Support Programmes (RCTS) publications increasing from 10 in 2004 to 49 in 2013 [66]. Lyle et al. [57] also noted that rural and remote academic centres substantially contributed to growing knowledge about rural and remote health. In a 2013 review of academic centres, it was reported that rural academics co-authored 363 peer-reviewed papers. In addition, these academic centres have established strong partnerships with health services, rural workforce agencies and other research centres and have supported rurally-based early career researchers and PhD students [57].

According to Sandhu et al. [59], through the expansion of medical training in underserved areas across the United States, there has been culturally and linguistic educational programmes to encourage recruitment and enrolment in clinical trials in rheumatic diseases especially lupus, where ethic/racial minorities are disproportionately affected by the condition. There have been other joint efforts with other National Institute of Health alongside Office of Research for Minority Health to address disparities in rheumatic diseases.

## Discussion

### Key findings

This narrative review found a wide-range of topics relating to the expansion of medical education in underserved areas and the impact on the future medical workforce, but there were few papers considering the actual ripple effects or long-term repercussions of a new medical school being set up. The main body of literature (*n* = 22; 61%) consisted of reviews of literature utilising heterogeneous review methodologies. After excluding two opinion / perspectives papers and eight review articles focused on policy, government strategy and workforce data, only four papers had collected original data. As a consequence, much of the literature reported on themes on one key concern around increasing medical undergraduates in underserved and/or rural contexts and recruiting and retaining the physician workforce.

There was a wealth of literature on medical students’ career choices, rural undergraduate medicine, rural exposure and placements, and tackling problems around the misdistribution of the healthcare workforce. This last issue therefore addressed our second research question around how new medical schools effect workforce issues in the local economy. There were a considerably smaller number of papers published (*n* = 10; 28%) with themes pertaining to the wider contribution of medical schools. This body of literature was rather less coherent, but nonetheless explored issues with respect to our first research question on the health, social, economic and research impacts of a new medical school in a region.

### How findings relate to other literature

Previous research exploring the wider health, social, economic and research impacts of new medical schools within a region are quite limited in the number of published studies. With respect to improvements in the health outcomes of local populations, the studies reviewed showed a positive impact with medical undergraduates immersing themselves into a community, better community engagement, provision of community-based medicine initiatives and an increase in access to healthcare to underserved groups. The literature showed that the impact of medical schools should not be limited to measureable health outcomes such as improvements in clinical care, but impact that looks at addressing non-clinical barriers to health [67]. Rodriguez et al. (2015) suggest that only 16% of health outcomes are related directly to clinical care, whereas the remaining 84% is accounted for by non-clinical factors such as health behaviours, social and economic factors and the physical environment, suggesting that clinical interventions alone do not improve health outcomes. The presence of a new medical school in itself will have limited impact on health equity, but medical schools that actively pursue a community engagement approach to generate ideas, adapt processes and create relationships between itself and the communities it serves help alleviate barriers to healthcare, as well as identify and implement interventions to improving health outcomes thereby mitigating against those barriers [67].

Studies synthesising the literature on the social accountability of medical schools and its measurement forms a small but discrete body of evidence, but the literature has shown that the conversion of a medical school’s social mission into action tend to be aspirational despite the existence of implementation frameworks. Boelen et al. (2019) has suggested that the social responsiveness of medical schools requires more explicit and quantifiable identification of health priority needs to ensure that when qualified, graduates acquire the desired outcomes and impacts to meet their social obligation. One possible avenue suggested by Boelen et al. (2019) is to link a medical school’s accreditation standards to foster positive social change, which are evaluated by teams visiting medical schools that are not only comprised of medical professionals, but representatives of other health stakeholders including patients and communities, therefore bringing in a wider spectrum of views on how schools fulfil their social obligation [68].

The findings on the economic contribution of new medical schools were limited to an article by Hogenbirk et al. [63] (2015) looking at one case study in Canada. The results suggested that there were direct impacts on income to the medical school itself, as well as increased total annual income to communities in the region, but more importantly new economic opportunities emerged. Due to the differences in economic modelling and the variation in country-specific contexts, comparing how the findings relate to other examples in the literature is problematic. A recent report assessing the establishment of an academic medical centre involving a partnership between Brown University and local health services in Rhode Island in the US provide an insight of how closer integration between the medical education sector and healthcare systems create significant economic and societal gains to the communities that live in a region. It was shown how an integrated medical centre had the potential for increases in statewide employment in healthcare, higher education and industry from 14.6% in 2020 to 18.6% by 2035. The biomedical sector also had the potential for growth from its current annual impact of $900 million in 2020 to $1.7 billion in 2035 [69]. The economic impact projections suggest significant transformative growth in employment, education and industry as a result of setting up an integrated academic medical centre.

The published literature showed that the impact of a new medical school or centre to a region has been limited to academic research activity. Despite the prospect and anticipation of research investment from local enterprise, pharmaceuticals and biomedical companies, this aspiration of large-scale industry financed research has yet to be fully realised. It was found that through the establishment of rural academic centres and increased medical training, there was a growth in an evidence base on remote rural health and diseases affecting underserved groups, increased publications, strong health research partnerships, regionally based PhD students and more opportunities for running studies and trials. Baquet et al. (2013) showed how a rural-community academic partnership model was developed between the University of Maryland School of Medicine (UMSOM), Eastern Shore Area Health Education Centre (ESAHEC) and the Office of Policy and Planning in the US, which increased the ability to jointly assess healthcare trends and needs, conduct joint research, undertake programme planning and evaluation and collaborate on grants. The rural-academic community partnership was equally important for developing research skills to conduct community partnered research and develop community cultural competence [70].

The findings from the vast majority of the literature reflect some of the immediate challenges of national governments and regional healthcare planners, with the magnitude of physician workforce shortages at the forefront of concern engulfing any further debates around how locally new medical schools can shift a region’s health, social, economic and research status. As shown in the literature, the reasons for medical staff shortages are multi-faceted with a large body of literature focusing on interventions addressing recruitment and retention. Much of the published literature overlooks the systemic concerns why doctors leave the workforce, instead the research focuses on meeting workforce shortages rather than investigating this issue at a more in-depth level. Taylor (2020) has argued that in the UK, the shortages will be alleviated in part by an increase in medical school numbers, but without a clear retention strategy, the Government will continue to lose doctors either by leaving the country or leaving the NHS altogether [71]. Taylor (2020) lists the key retention factors (including salary, contract, inflexible rotas to name a few) but fundamentally indicates there is little known evidence why doctors leave, and without an understanding of such issues, the UK Government is ill-equipped to grapple with alleviating the problems altogether [71]. The issue of workforce recruitment and retention may be addressed in part by an overall increase in student numbers, but medical schools must partner with local healthcare systems, so that their graduates once qualified, will explore local employment opportunities and consider what they find are creative and attractive working conditions to sustain an effective healthcare model [68]. Even if better coordination between medical schools and the local healthcare system are in place to ensure that graduates remain regionally based, the Queensland example in Australia has shown that there is a disconnect between undergraduate and postgraduate specialty training. Therefore, even after completion of undergraduate medical education, efforts must be taken to develop programmes in postgraduate specialty training that are regionally based to retain the newly trained medical workforce [72].

This narrative review has shown that there are a much wider range of factors that need to be taken into account when assessing the impact of new medical schools, with particular consideration being given to a region’s health, social, economic and research dynamics. It should be noted that impacts on health care quality and economic investment are notoriously difficult to measure, therefore it is essential for medical schools to capture and demonstrate impact through value-added case studies [73] and impact evaluations [74]. Despite the difficulties in evidencing impact, the UK’s Medical Schools Council have highlighted the contributions medical schools have made through a series of case studies on how medical school researchers have helped to improve outcomes for patients and changed the way medicine is practiced for the benefit of patients [73]. Furthermore, medical schools have been involved in starting new companies, supporting well established organisations, and created jobs through their engagement with the life sciences sector helping to generate income, at the time of publication in 2015, of more than £50 billion of turnover. Therefore, developing an evaluative framework that moves away from the social accountability model towards examining contributions from the life sciences sector and from research infrastructure investments, will aid in demonstrating how medical schools can drive innovation in healthcare research, encourage economic growth around research clusters and lead to the development of a highly-skilled workforce across academia, industry and the NHS in a region [75].

### Limitations

When undertaking this narrative review there were a number of limitations that were encountered. The lack of established published methodological approaches to a narrative review with respect to extracting and analysing data made it a challenge to define what to extract from each paper. Despite using guidance from Ferrari (2015) [27] who recommended extracting data from the (1) key results, (2) quality of the results obtained and (3) interpretation of results, these three criteria were difficult to implement in practice. We collapsed all three results criteria grouping them under one ‘results’ column heading. The articles included were restricted to papers published in English which may have overlooked literature published in non-English language journals. The papers reviewed were evaluated by only one reviewer, which may have biased the data extracted and the analysis. Furthermore, the literature reviewed largely was heterogeneous and as a consequence the themes identified varied considerably, and also made it challenging to draw out any common themes and conclusions. Lastly, much of the literature drew heavily from studies on undergraduate medical education and physician recruitment and retention concerns, which skewed the results focusing mainly on workforce issues.

### Implications for research, education, practice and policy

Further research is needed to determine with far greater insight and complexity the direct and indirect consequences of new medical schools being established in a region. We have outlined what the possible impacts could encompass helping to kick-start new research in the field with a view to evaluate the effects on health, social, economic and research activity. Assessing and evaluating the influence of new medical schools in one region would offer local insight, but expanding the scope to a national evaluation in particular in England with its five new medical schools would provide valuable evidence based research for understanding how long it takes for social and economic impacts to be felt in a region and what are the implication on the health outcomes of the local population long-term. The preparatory work informing a larger study on the impact of a new medical school in Kent and Medway has commenced with the completion of an evaluability assessment (unpublished) by the authors (FH, CM and SP).

## Conclusion

We have described what impacts new medical schools and increased opportunities for medical training may contribute to a region, both in terms of understanding the types of medical undergraduates likely to practice in primary care and community-based medicine, in areas located in rural and underserved settings. The studies included in the review found a wealth of information on a wide-range of topics on the expansion of undergraduate education and its implications on the future medical workforce. It was shown that medical schools can have a positive effect on the health, social, economic and research activity of a region, but this literature tended to be heterogeneous in focus without any consideration of the inter-connections between the wider societal and economic impacts arising from long-term sustainable change being brought to a region. Despite the limited evidence base, there were positive health outcomes on local populations with medical students engaging with communities and improving access to healthcare, aspirations for enhancing the social accountability of medical schools to reduce health disparities, evidence of newer economic opportunities arising from actual and indirect expenditures associated with a new medical school, and the prospect and anticipation of new research investment into a region. The findings from this review indicate the impacts of new medical schools extend beyond workforce issues (whilst acknowledging they should remain a high agenda priority), nonetheless, there is a growing need to look beyond immediate concerns and towards wider trends informing future research on direct and inconsequential impacts involving key stakeholders such as national and local government, the business and innovations sector, community partnerships and the medical schools themselves to determine regional and national policy directions.

## Supplementary Information

**Additional file 1: Table S1.** Medline search strategy.

**Additional file 2: Table S2.** Summary table of papers reviewed.

## Data Availability

The datasets used and/or analysed during the current study are available from the corresponding author.
