# Supplementary material for: What are the impacts of setting up new medical schools? A narrative review
Source: BMC Med Educ. 2022 Nov 7;22:759. doi: 10.1186/s12909-022-03835-4 (PMC9639304; doi:10.1186/s12909-022-03835-4)
Supplement: Supplementary file 1 — Additional file 1: Table S1. Medline search strategy. [file 12909_2022_3835_MOESM1_ESM.docx]

**Table S1: Medline search strategy**

Database(s): **Ovid MEDLINE(R)**1946 to February Week 2 2021

| **#** | **Searches** | **Results** |
| --- | --- | --- |
| 1 | Schools, Medical/ | 25974 |
| 2 | (new medical school* or medical school* or school* of medicine or clinical training).ti,ab. | 41159 |
| 3 | 1 or 2 | 55836 |
| 4 | exp Medically Underserved Area/ | 7122 |
| 5 | medically underserved.ti,ab. | 1243 |
| 6 | Rural Population/ | 61415 |
| 7 | Rural Health Services/ | 13061 |
| 8 | (local communit* or rural communit*).ti,ab. | 15910 |
| 9 | Professional Practice Location/ | 2987 |
| 10 | Social Responsibility/ | 19560 |
| 11 | (social accountabilit* or social responsibilit*).ti,ab. | 1742 |
| 12 | (economic impact* or local economy or economic benefit*).ti,ab. | 13340 |
| 13 | (community benefit* or community impact* or local impact*).ti,ab. | 987 |
| 14 | Health Workforce/ | 13428 |
| 15 | Employment/ | 46868 |
| 16 | (healthcare workforce or health workforce or medical workforce or workforce recruitment).ti,ab. | 3478 |
| 17 | General Practice/ | 13694 |
| 18 | (general practice or general practitioner* or GPs or doctor* or hub*).ti,ab. | 205078 |
| 19 | Population Health/ | 1025 |
| 20 | 4 or 5 or 6 or 7 or 8 or 9 or 10 or 11 or 12 or 13 or 14 or 15 or 16 or 17 or 18 or 19 | 384135 |
| 21 | 3 and 20 | 6350 |
| 22 | exp Developing Countries/ | 76079 |
| 23 | 21 not 22 | 6241 |
| 24 | limit 23 to (yr="2011 - 2021" and (english or french) and (government publication or meta analysis or "review" or "systematic review")) | 138 |
