# Supplementary material for: What are the impacts of setting up new medical schools? A narrative review
Source: BMC Med Educ. 2022 Nov 7;22:759. doi: 10.1186/s12909-022-03835-4 (PMC9639304; doi:10.1186/s12909-022-03835-4)
Supplement: Supplementary file 2 — Additional file 2: Table S2. Summary table of papers reviewed. [file 12909_2022_3835_MOESM2_ESM.docx]

**Table S2: Summary table of papers reviewed**

| **Author(s)** | **Type of study** | **Methods to test hypothesis** | **Country** |
| --- | --- | --- | --- |
| Amin M, Chande S, Park S, Rosenthal J, Jones M (2018) | Rapid evidence appraisal | Pragmatic rapid appraisal of literature on UG influences on GP career choices | UK |
| Asghari S,Kirkland MC, Blackmore J, Boyd S, Farrell A, Rourke J, et al (2020) | Systematic review of reviews | Systematic review of reviews on recruitment and retention of family physicians in rural areas; to establish an overall conclusion and provide a set of fundamental factors | Canada |
| Avery Jr. DM, Wheat JR, Leeper JD, McKnight JT, Ballard BG, Chen J (2012) | Cross-sectional survey | Questionnaire to study the ability of admission factors to predict family medicine specialty choice | USA |
| Bailey JK, Mendis K, Dutton T, Stevens W, McCrossin T (2015) | PubMed Review | PubMed review to assess the contribution of Rural Clinical Training & Support Programmes (RCTS) to rural health research | Australia |
| Barber C, van der Vleuten C, Leppink J, Chahine S (2020) | Narrative review | Narrative review aimed to identify and document common themes and indicators across a social accountability framework | Canada |
| Barrett FA, Lipsky MS, Lutfiyya MN (2011) | Critical review | Critical review of North American studies on student outcomes associated with rural training experiences | USA |
| Budhathoki SS, Zwanikken PA, Pokharel PK, Scherpbier AJ (2017) | Systematic review | Systematic review conducted to identify influences on medical students’ motivations to work in rural areas in low and middle-income countries | Nepal |
| Danish A, Blais R, Champagne F (2019) | Review article | N/A  Review article to examine the strategic relevance of interventions identifying and prioritising determinants of physician shortages and analysing the intervention based on their ability to target the determinants | Canada |
| Darbyshire D, Gordon M, Baker P, Agius S, McAleer S (2019) | Systematic review | Systematic review of the evidence on interventions that encourage careers in academic medicine | UK |
| Edelman A, Taylor J, Ovseiko P V, Topp SM (2017) | Systematic review | Systematic review to synthesise and critically appraise the evidence on the role of academic health centres (AHCs) in improving health equity | Australia |
| Ellaway RH, Malhi R, Bajaj S, Walker I, Myhre D (2018) | Scoping review | Critical scoping review of the connections between the social mission and medical school admissions | Canada |
| Fancher TL, Keenan C, Meltvedt C, Stocker T, Harris T, Morfin J, et al (2011) | Review article | N/A  Provides an overview of the Transforming Education and Community Health (TEACH) Programme, including curriculum details, outcomes, barriers to continue and expanded implementation | USA |
| Farmer J, Kenny A, McKinstry C, Huysmans RD (2015) | Scoping review | Scoping review to map the existing evidence on the relationship between professional entry-level pre-vocational medical education delivered in rural settings and rural workforce outcomes | Australia |
| Goodfellow A, Ulloa JG, Dowling PT, Talamantes E, Chheda S, Bone C, et al (2016) | Systematic literature review | Systematic review of peer reviewed studies | USA |
| Grobler L, Marais BJ, Mabunda S (2015) | Systematic review | Systematic review to assess the effectiveness of interventions aimed at increasing the proportion of health professionals working in rural and other underserved areas | South Africa |
| Guilbault RW, Vinson JA (2017) | Systematic review and meta-analysis | Qualitative and quantitative summary of existing evidence that UG medical student clinical education in rural or underserved areas also improves the likelihood of eventual practice of primary care specialties in those areas | USA |
| Hogenbirk JC, Robinson DR, Hill ME, Pong RW, Minore B, Adams K, et al (2015) | Quantitative study | Quantitative study to assess the economic contribution of Northern Ontario School of Medicine (NOSM) to northern Ontario communities participating in the medical education programme | Canada |
| Holst J, Normann O, Herrmann M (2015) | Mixed methods study | Mixed methods evaluation the Magdeburg rural elective aimed at enhancing physician recruitment and retention in rural practice | Germany |
| Holst J (2020) | Integrative review | Integrative review assesses the effects of rural placements during UG medical training on graduates’ likelihood to take up rural practice | Germany |
| Hurst S (2014) | Opinion piece | N/A  Efforts to increase medical graduates in rural areas by providing training in rural medicine in fact actively discourages them from such practice | Switzerland |
| Julian K, Riegels NS, Baron RB (2011) | Perspectives piece | N/A  Examines the impact of educational interventions in UG medical education om primary care carer choices and makes suggestions for future educational change | USA |
| Kirch DG, Henderson MK, Dill MJ (2012) | Review article | N/A  Review article to understand what lessons workforce projections can teach and start to find solutions to meet the demand | USA |
| Lyle D, Greenhill J (2018) | Review article | N/A  Reports on the contribution of University Department of Rural Health (UDRHs) and Rural Clinical Schools (RCSs) in developing rural health and the rural health workforce; contribute to the rural clinical academic workforce | Australia |
| Matsumoto M, Takeuchi K, Yokobayashi K, Tazuma S (2015) | Review article | N/A  Review paper summarises past literature and proposes a future strategy to deal the skewed distribution of physicians in Japan | Japan |
| Myhre DL, Bajaj S, Jackson W (2015) | Scoping review | Scoping review focusing on factors that predispose urban-origin students to choose a career in rural medicine | Canada |
| Ogden J, Preston S, Partanen RL, Ostini R, Coxeter P (2020) | Systematic review & meta-analysis | Systematic review and meta-analysis of the effects of rural pipeline factors on the likelihood of later general practice in rural medicine | Australia |
| O’Sullivan BG, McGrail MR, Russell D, Chambers H, Major L (2018) | Scoping review | Scoping review to describe the characteristics and outcomes of rural immersion programmes implemented in Australian medical schools | Australia |
| Palsdottir B, Barry J, Bruno A, Barr H, Clithero A, Cobb N, et al (2016) | Review article | N/A  Review article providing an overview of six strategies and interventions that provide context-relevant health professional education within the health system | USA |
| Parlier AB, Galvin SL, Thach S, Kruidenier D, Fagan EB (2018) | Narrative review | Narrative review of literature on individual, educational and professional characteristics of physicians | USA |
| Puschel K, Rojas P, Erazo A, Thompson B, Lopez J, Barros J (2014) | Qualitative literature review and mixed methods | Literature review and mixed methods used to study the significance and relevance of social responsibility in the academic training of medical schools in Latin America | Chile |
| Putri LP, O’Sullivan BG, Russell DJ, Kippen R (2020) | Scoping review | Scoping review to understand the factors associated with doctors working in rural areas to identify effective strategies to improve the rural medical workforce in Asia-Pacific LMICs | Indonesia |
| Sandhu VK, Jose DM, Feldman CH (2020) | Review article | N/A  Explores rheumatology, education and work in underserved areas and issues of accessing healthcare | USA |
| Stagg P, Prideaux D, Greenhill J, Sweet L (2012) | Systematic review | Systematic review to understand if and how medical students’ career choices are influenced by with interactions with preceptors (i.e. a clinician with whom the medical student is working) | Australia |
| Strasser R (2016) | Review article | N/A  Explores the remote rural context in healthcare and the educational programmes provided in these settings | Canada |
| Thistlethwaite JE, Bartle E, Chong AA, Dick ML, King D, Mahoney S, et al (2013) | Literature review | Review to explore, analysis and synthesise evidence on the effectiveness of longitudinal placements for medical students to determine which aspects are critical to successful outcomes | Australia |
| Verma P, Ford JA, Stuart A, Howe A, Everington S, Steel N (2016) | Systematic review | Systematic review to evaluate interventions and strategies used to recruit and retain primary care doctors internationally | UK |
